# Dr. Donald F. McPherson

**Published:** 1886-04

**Authors:** 


### Dr. Donald F. McPherson.

The profession in this city are peculiarly unfortunate in the loss of one of the best educated of their younger physicians. After a distressing illness of several weeks, the Doctor died at his former home in Munford, N. Y. He was in every way qualified to fill a prominent place in the profession, and was fast acquiring a reputation for himself. He was a lecturer in the Buffalo Medical College; until recently, a Sanitary Inspector for the Board of Health. He was a member of the Buffalo Medical Club and the Physicians' Club of this city. Both organizations passed appropriate resolutions expressing their deep sorrow at his untimely death, and tendering their heartfelt sympathy to the bereaved widow.

---

In our last number we omitted to mention that Dr. E. C. W. O'Brien, of this city, and Dr. Clark, of Niagara Falls, have been appointed, by the Council, curators in the Buffalo Medical College. The selections are excellent, and no doubt both will fill with honor this important office.

---

The sad news of the death of our friend and colleague Prof. C. C. F. Gay is brought to us as we are going to press. In our next issue we will give a full account of his life and labors.

---

Through the courtesy of the *Buffalo Morning Express*, we are able to present to our readers the excellent portrait of the late Professor Austin Flint.

